# Supplementary material for: Field performance and cost‐effectiveness of a point‐of‐care triage test for HIV virological failure in Southern Africa
Source: J Int AIDS Soc. 2023 Oct 6;26(10):e26176. doi: 10.1002/jia2.26176 (PMC10558896; doi:10.1002/jia2.26176)

**Supplementary Figure 1 Legend**

Current antiretroviral therapy (ART) monitoring algorithm for people living with HIV in Mozambique. Adapted from the updated 2021 Mozambican Guidelines on HIV prevention, testing, treatment, service delivery and monitoring. EAC: enhanced adherence counselling.


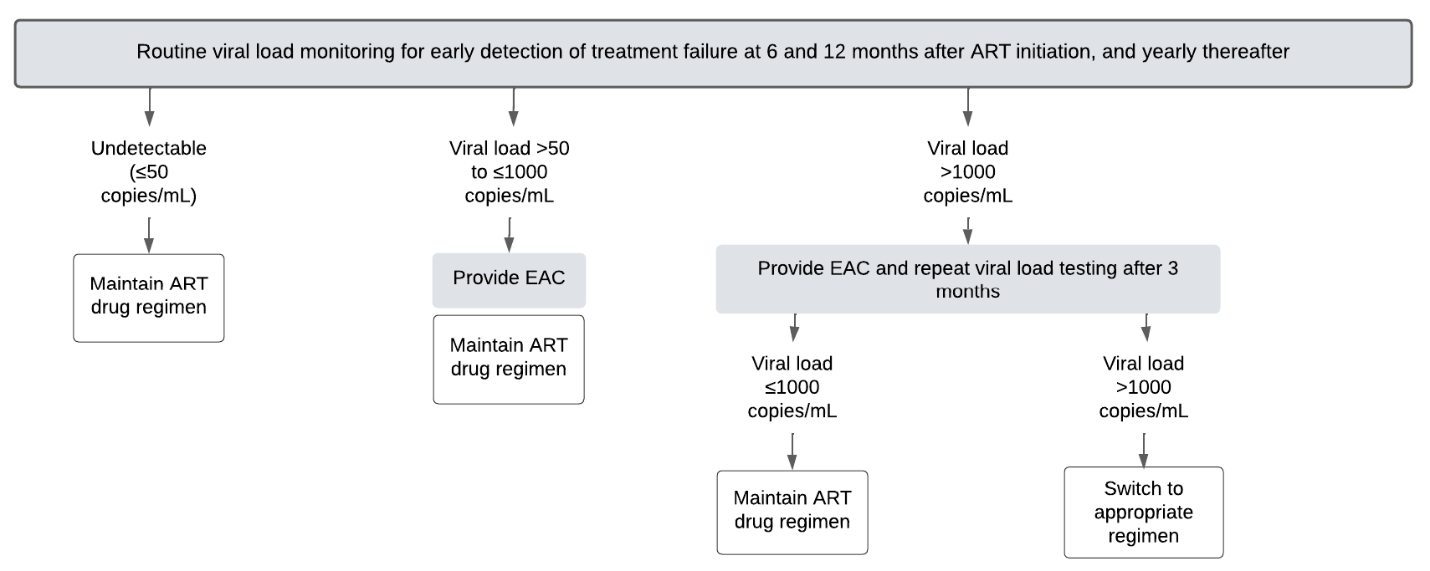


**Supplementary Figure 2 Legend**

Three different proposed algorithms where the IP-10 lateral flow assay (LFA) may be integrated combined with viral load (VL) confirmatory testing for antiretroviral therapy (ART) monitoring. These strategies are based on the current Mozambican ART monitoring guidelines and they mainly differ for the timing to perform the confirmatory VL test after a positive IP-10 result, and the use of the IP-10 LFA as a second VF screening at the 3-month control among IP-10 positive subjects: 2a) VL test performed immediately after an IP-10 LFA positive result; 2b) VL test performed 3 months after an IP-10 LFA positive result and after receiving enhanced adherence counselling (EAC); 2c) VL test performed after two IP-10 LFA positive results, of which the second one is obtained 3 months after the first IP-10 LFA positive result and after receiving EAC.


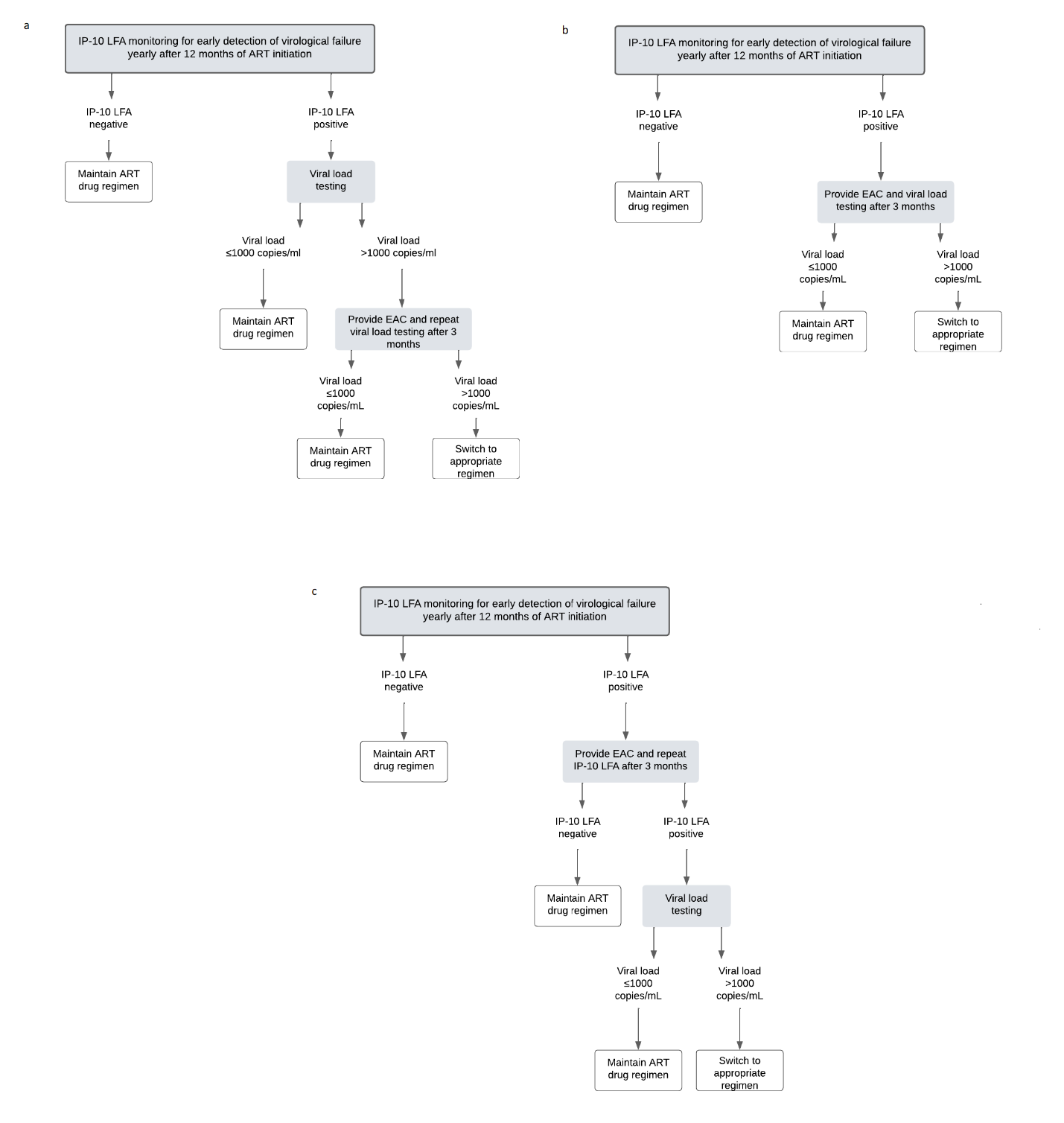


**Supplementary Figure 3 Legend**

Accuracy of IP-10 model for identifying PLHIV with virological failure (VF) according to the observed prevalence of individuals on antiretroviral therapy (ART) with VF. 3a) Positive predictive value (PPV) estimated for sensitivity=92% and 3 different specificity scenarios according to the estimated confidence interval (Sp=35%; 95%CI, 28-43%). 3b) Negative predictive value (NPV) estimated for specificity=35% and 3 different sensitivity scenarios according to the estimated confidence interval (Se=92%; 95%CI, 78-98%). Dashed/dotted line indicates PPV and NPV at the prevalence of VF observed in the study (17.7%).

**
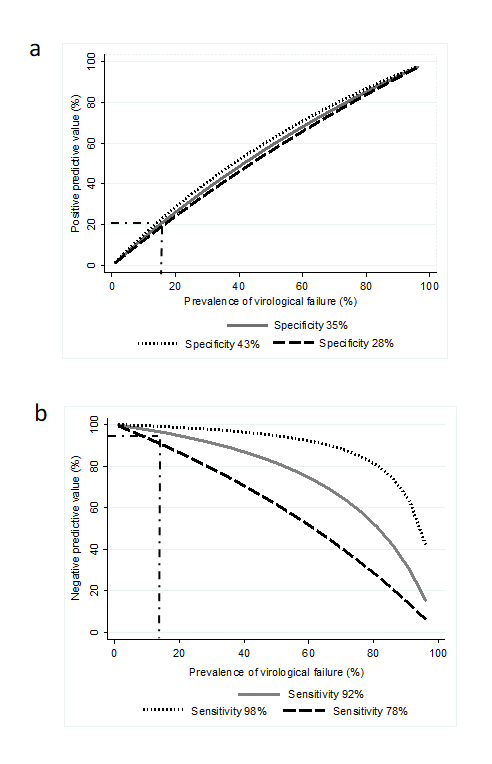
**

**Supplementary Figure 4 Legend**

Correlations between ELISA IP-10 measurements (pg/mL) and IP-10 lateral flow assay (LFA) reading values (arbitrary units, Cube Reader) using: 4a) plasma samples (209), 4b) finger prick capillary blood samples (208). The R-squared (R2) values for fit to the linear regression line (continuous line) are indicated. IP-10 values were log-transformed for a better data visualization.


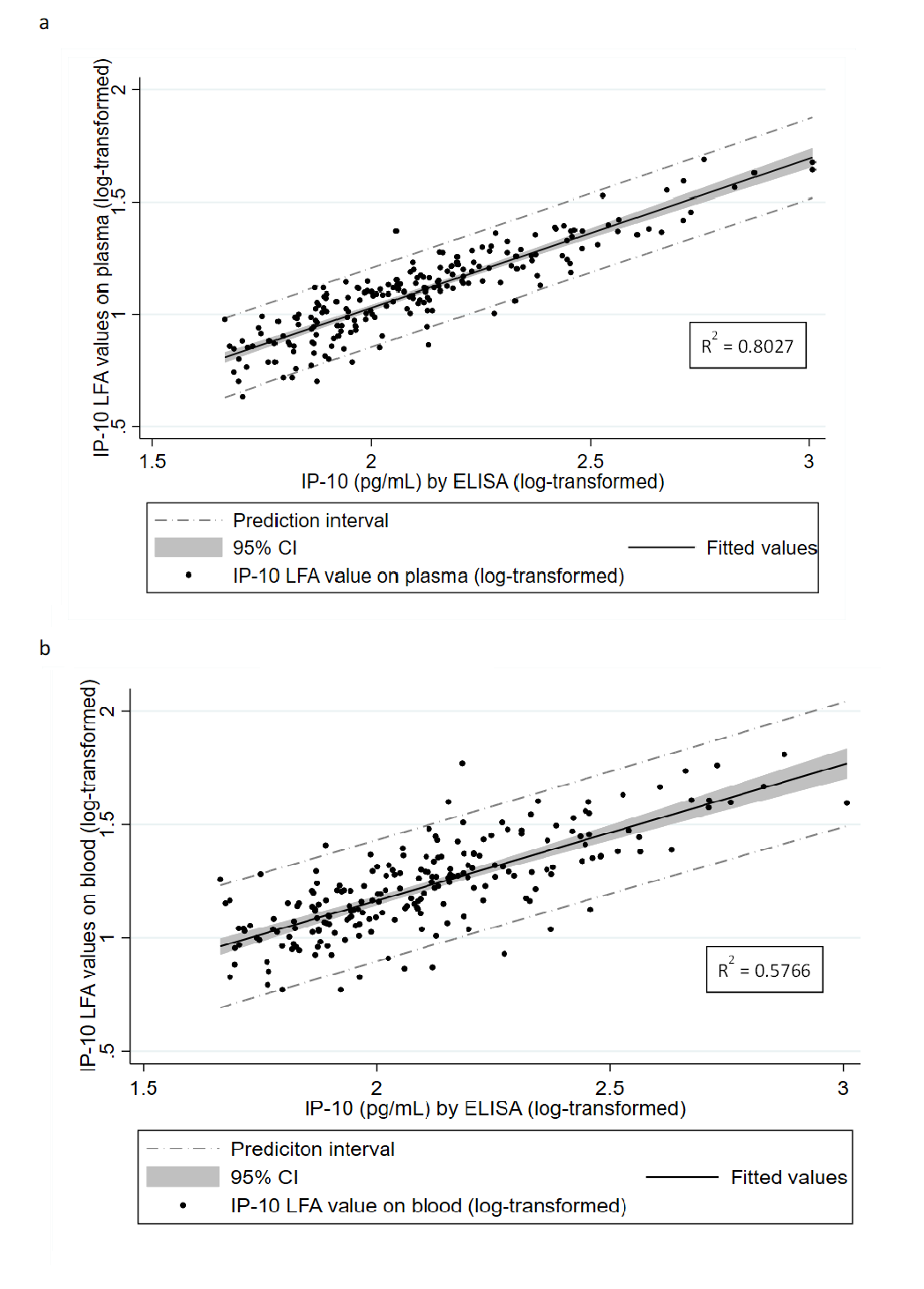

Supplement: Supplementary file 1 — Figure S1: Current antiretroviral therapy (ART) monitoring algorithm for people living with HIV in Mozambique. Adapted from the updated 2021 Mozambican Guidelines on HIV prevention, testing, treatment, service delivery and monitoring. EAC: enhanced adherence counselling. Figure S2: Three different proposed algorithms where the IP‐10 lateral flow assay (LFA) may be integrated combined with viral load (VL) confirmatory testing for antiretroviral therapy (ART) monitoring. These strategies are based on the current Mozambican ART monitoring guidelines and they mainly differ for the timing to perform the confirmatory VL test after a positive IP‐10 result, and the use of the IP‐10 LFA as a second VF screening at the 3‐month control among IP‐10 positive subjects: 2a) VL test performed immediately after an IP‐10 LFA positive result; 2b) VL test performed 3 months after an IP‐10 LFA positive result and after receiving enhanced adherence counselling (EAC); 2c) VL test performed after two IP‐10 LFA positive results, of which the second one is obtained 3 months after the first IP‐10 LFA positive result and after receiving EAC. Figure S3: Accuracy of IP‐10 model for identifying PLHIV with virological failure (VF) according to the observed prevalence of individuals on antiretroviral therapy (ART) with VF. 3a) Positive predictive value (PPV) estimated for sensitivity=92% and 3 different specificity scenarios according to the estimated confidence interval (Sp=35%; 95%CI, 28‐43%). 3b) Negative predictive value (NPV) estimated for specificity=35% and 3 different sensitivity scenarios according to the estimated confidence interval (Se=92%; 95%CI, 78‐98%). Dashed/dotted line indicates PPV and NPV at the prevalence of VF observed in the study (17.7%). Figure S4: Correlations between ELISA IP‐10 measurements (pg/mL) and IP‐10 lateral flow assay (LFA) reading values (arbitrary units, Cube Reader) using: 4a) plasma samples (209), 4b) finger prick capillary blood samples (208). The R‐squared [file JIA2-26-e26176-s002.docx]
