# Supplementary material for: Field performance and cost‐effectiveness of a point‐of‐care triage test for HIV virological failure in Southern Africa
Source: J Int AIDS Soc. 2023 Oct 6;26(10):e26176. doi: 10.1002/jia2.26176 (PMC10558896; doi:10.1002/jia2.26176)
Supplement: Supplementary file 3 — Table S2: Socio‐demographic and clinical characteristics associated with virological failure (VF) (viral load >1,000 copies/mL) among study participants (n=209). Crude odds ratios from logistic regression analyses. [file JIA2-26-e26176-s004.docx]

**Supplementary Table 2. Socio-demographic and clinical characteristics associated with virological failure (VF) (viral load >1,000 copies/mL) among study participants (n=209). Crude odds ratios from logistic regression analyses.**

|  | **N (%) or median (IQR)** | | | | | | | | | | | | **cOR** | | **95%CI** | | **p-value** | |
| --- | --- | --- | --- | --- | --- | --- | --- | --- | --- | --- | --- | --- | --- | --- | --- | --- | --- | --- |
|  | **Total** | | | | **VL≤1000 copies/mL** | | | | **VL>1000 copies/mL** | | | |  | |  | |  | |
|  | **(n=209) ^a^** | | | | **(n=171)** | | | | **(n=37)** | | | |  |  |  |  |  |  |
| **Biological sex** |  | |  | |  | |  | |  | |  | |  | |  | |  | |
| Male | 34 | | (16.35) | | 28 | | (16.28) | | 6 | | (16.22) | | Ref. | |  | |  | |
| Female | 175 | | (83.73) | | 144 | | (83.72) | | 31 | | (83.78) | | 0.96 | | 0.38-2.43 | | 0.925 | |
| **Age (years)** | 38 | | (31-44) | | 37 | | (31-44) | | 39 | | (34-46) | | 1.03 | | 0.98-1.07 | | 0.246 | |
| **Employment** |  | |  | |  | |  | |  | |  | |  | |  | |  | |
| Employed | 43 | | (20.57) | | 38 | | (22.09) | | 5 | | (13.51) | | Ref. | |  | |  | |
| Part-time | 21 | | (10.10) | | 14 | | (8.14) | | 7 | | (18.92) | | 3.62 | | 1.03-12.70 | | **0.045** ^b^ | |
| Not employed | 145 | | (69.38) | | 120 | | (69.77) | | 25 | | (67.57) | | 1.48 | | 0.55-3.99 | | 0.437 | |
| **Marital status** |  | |  | |  | |  | |  | |  | |  | |  | |  | |
| Married ^c^ | 58 | | (27.75) | | 51 | | (29.65) | | 7 | | (18.92) | | Ref. | |  | |  | |
| Single | 139 | | (66.51) | | 112 | | (65.12) | | 27 | | (72.97) | | 1.68 | | 0.70-4.02 | | 0.244 | |
| Divorced | 7 | | (3.35) | | 7 | | (4.07) | | 0 | | (0.00) | | 0.46 | | 0.02-8.86 | | 0.605 | |
| Widowed | 5 | | (2.39) | | 2 | | (1.16) | | 3 | | (8.11) | | 9.61 | | 1.60-57.85 | | **0.013** ^b^ | |
| **Educational level** |  | |  | |  | |  | |  | |  | |  | |  | |  | |
| No primary school | 10 | | (4.78) | | 6 | | (3.49) | | 4 | | (10.81) | | Ref. | |  | |  | |
| Primary school | 135 | | (64.59) | | 112 | | (65.12) | | 23 | | (62.16) | | 0.30 | | 0.08-1.09 | | 0.067 ^b^ | |
| Secondary school | 58 | | (27.75) | | 49 | | (28.49) | | 9 | | (24.32) | | 0.28 | | 0.07-1.11 | | 0.070 ^b^ | |
| Post-secondary school | 2 | | (0.96) | | 1 | | (0.58) | | 1 | | (2.70) | | 1.44 | | 0.11-18.73 | | 0.779 | |
| University | 4 | | (1.91) | | 4 | | (2.33) | | 0 | | (0.00) | | 0.16 | | 0.01-3.78 | | 0.256 | |
| **BMI** ^d^ (n=202) |  | |  | |  | |  | |  | |  | |  | |  | |  | |
| Normal weight | 58 | | (28.71) | | 46 | | (27.88) | | 12 | | (32.43) | | Ref | |  | |  | |
| Underweight | 2 | | (0.99) | | 1 | | (0.61) | | 1 | | (2.70) | | 3.72 | | 0.35-38.92 | | 0.273 | |
| Overweight | 63 | | (31.19) | | 50 | | (30.30) | | 13 | | (35.14) | | 0.99 | | 0.42-2.37 | | 0.990 | |
| Obesity | 79 | | (39.11) | | 68 | | (41.21) | | 11 | | (29.73) | | 0.62 | | 0.26-1.51 | | 0.296 | |
| **Previous confirmed COVID-19 diagnosis** | 7 | | (3.35) | | 5 | | (2.91) | | 2 | | (5.41) | | 2.14 | | 0.46-9.99 | | 0.331 | |
| **Previous COVID-19 hospitalization** | 1 | | (0.48) | | 1 | | (0.58) | | 0 | | (0.00) | | 1.52 | | 0.06-38.16 | | 0.797 | |
| **Pregnancy** (n=175) |  | |  | |  | |  | |  | |  | |  | |  | |  | |
| No or unknown ^e^ | 170 | | (97.14) | | 141 | | (97.92) | | 29 | | (93.55) | | Ref. | |  | |  | |
| Yes | 5 | | (2.86) | | 3 | | (2.08) | | 2 | | (6.45) | | 3.43 | | 0.64-18.21 | | 0.149 | |
| **Hypertension** | 22 | | (10.53) | | 17 | | (9.88) | | 5 | | (13.51) | | 1.50 | | 0.54-4.21 | | 0.438 | |
| **Diabetes** | 3 | | (1.44) | | 2 | | (1.16) | | 1 | | (2.70) | | 2.80 | | 0.36-21.89 | | 0.326 | |
| **Hepatitis B** (n=65) | 3 | | (4.62) | | 1 | | (2.44) | | 2 | | (8.33) | | 3.00 | | 0.37-24.23 | | 0.303 | |
| **Epilepsy** | 3 | | (1.44) | | 2 | | (1.16) | | 1 | | (2.70) | | 2.80 | | 0.36-21.89 | | 0.326 | |
| **Symptoms at the study visit ^f^** | 4 | | (1.91) | | 3 | | (1.74) | | 1 | | (2.70) | | 1.99 | | 0.28-13.93 | | 0.488 | |
| **Current TB** | 1 | | (0.48) | | 0 | | (0.00) | | 1 | | (2.70) | | 14.18 | | 0.57-355.02 | | 0.107 | |
| **Previous or current TB treatment** | 51 | | (24.40) | | 39 | | (22.67) | | 12 | | (32.43) | | 1.66 | | 0.77-3.56 | | 0.195 | |
| **WHO stage at ART initiation** |  | |  | |  | |  | |  | |  | |  | |  | |  | |
| Stage 1 or 2 | 164 | | (78.47) | | 136 | | (79.07) | | 28 | | (75.68) | | Ref. | |  | |  | |
| Stage 3 or 4 | 45 | | (21.53) | | 36 | | (20.93) | | 9 | | (24.32) | | 1.25 | | 0.55-2.83 | | 0.598 | |
| **WHO stage at the study visit** | |  | |  | |  | |  | |  | |  | |  | |  | |  |
| Stage 1 or 2 | 159 | | (76.08) | | 132 | | (76.74) | | 27 | | (72.97) | | Ref. | |  | |  | |
| Stage 3 or 4 | 50 | | (23.92) | | 40 | | (23.26) | | 10 | | (27.03) | | 1.25 | | 0.57-2.76 | | 0.583 | |
| **IP-10 LFA reading ^g^** (n=208) | 16.2 | | (11.9-22.8) | | 14.6 | | (11.3-20.0) | | 24 | | (17.6-36.3) | | 2.38 ^h^ | | 1.66-3.40 | | **<0.001** | |
| **Days since last VL** | 7 | | (4-14) | | 8 | | (5-14) | | 6 | | (2-12) | | 0.97 | | 0.93-1.02 | | 0.233 | |
| **Years since ART initiation** | 6.5 | | (4.2-9.4) | | 6.33 | | (4.2-9.0) | | 7.50 | | (4.8-11.1) | | 1.09 | | 0.99-1.20 | | 0.072 | |
| **Years since HIV diagnosis** | 7 | | (4-10) | | 6 | | (4-9) | | 8 | | (5-12) | | 1.11 | | 1.01-1.22 | | **0.026** | |
| **Missed ART ^i^** |  | |  | |  | |  | |  | |  | |  | |  | |  | |
| None | 182 | | (87.08) | | 158 | | (91.86) | | 24 | | (64.86) | | Ref. | |  | |  | |
| At least once dose a month | 27 | | (12.92) | | 14 | | (8.14) | | 13 | | (35.14) | | 6.02 | | 2.56-14.16 | | **<0.001** | |
| **Current ART regimen** |  | |  | |  | |  | |  | |  | |  | |  | |  | |
| TDF+3TC+DTG | 174 | | (83.25) | | 150 | | (87.21) | | 24 | | (64.86) | | Ref. | |  | |  | |
| TDF+FTC+EFV | 21 | | (10.05) | | 14 | | (8.14) | | 7 | | (18.92) | | 3.18 | | 1.19-8.46 | | **0.021** | |
| Others | 14 | | (6.70) | | 8 | | (4.65) | | 6 | | (16.22) | | 4.70 | | 1.55-14.21 | | **0.006** | |

Legend of table:

^a^ For those variables with missing values, the total number of observations included is indicated next to the name of the variable.

^b^ This category was not included in the multivariable analysis because of the small n and because it has not the potential to be a factor included in the ART monitoring algorithm.

^c^ This category includes married, civil union or legal partnership.

^d^ BMI ranges: normal weight: 18.5-24.9 kg/m2, underweight: <18.5 kg/m2, overweight: 25-29.9 kg/m2; obesity: ≥30 kg/m2.

^e^ All of them were unknown, except one.

^f^ After COVID-19 screening, symptoms assessed were fever, night sweats, myalgia, fatigue, headache, diarrhoea and skin rash

^g^ IP-10 LFA values were log-transformed for a better adjustment of skewed data.

^h^ For OR calculation, IP-10 reading values were categorized by increments of 10-units. Therefore, an increase of 10 units in the IP-10 reading value corresponds to a 2.38 OR of having VF.

^i^ Self-reported ART adherence.

| Abbreviations: 3TC: Lamivudine, ART: antiretroviral therapy, BMI: body mass index, CI: confidence interval, cOR: crude odds ratio, DTG: dolutegravir, FTC: emtricitabine, EFV: efavirenz, IQR: interquartile range, LFA: lateral flow assay, Ref: reference category, TB: tuberculosis, TDF: tenofovir, VL: viral load. |
| --- |
